# Factors predictive of papillary thyroid micro-carcinoma with bilateral involvement and central lymph node metastasis: a retrospective study

**DOI:** 10.1186/1477-7819-10-67

**Published:** 2012-05-19

**Authors:** Yi-Li Zhou, Er-li Gao, Wei Zhang, Han Yang, Gui-Long Guo, Xiao-Hua Zhang, Ou-Chen Wang

**Affiliations:** 1 Department of Oncology, The First Affiliated Hospital of Wenzhou Medical College, No.2 Fuxue lane, Wenzhou, Zhejiang, China

**Keywords:** Papillary micro-carcinoma, Bilateral thyroid cancer, Multifocality, Thyroidectomy, Central lymph node metastasis

## Abstract

**Background:**

The optimal resection extent for papillary thyroid microcarcinoma (PTMC) remains controversial. The objective of the study was to investigate risk factors of bilateral PTMC and central lymph node metastasis (CLNM) to guide surgical strategies for PTMC patients.

**Methods:**

We retrospectively reviewed 211 PTMC patients who underwent total thyroidectomy (TT) and 122 clinical lymph node-negative (cN0) cases that underwent prophylactic central lymph node dissection (CLND) between 2010 and 2011. The frequency, pattern, and predictive factors for bilateral PTMC and CLNM in these patients were studied using univariate and multivariate analysis with respect to the following variables: age, gender, extrathyroidal extension (ETE), T stage, with Hashimoto thyroiditis (HT), tumor size and multifocality based on final pathology, and preoperative evaluation using ultrasonography (US).

**Results:**

Fifty-four of 211 (25.6%) patients had bilateral PTMC. In multivariate analysis, multifocality (*P* < 0.001, OR = 23.900) and tumor size ≥7 mm (*P* = 0.014, OR = 2.398) based on US were independent predictive factors for bilateral PTMC which was also independently associated with multifocality (*P* < 0.001, OR = 29.657) and tumor size ≥7 mm (*P* = 0.005, OR = 2.863) based on final pathology. Among 122 cN0 patients who underwent prophylactic CLND, we found 49.2% of patients had CLNM. CLNM was independently associated with men, age <50 years and tumor size ≥7 mm based on final pathology or preoperative US.

**Conclusions:**

TT should be considered for PTMC patients who are found multifocality and tumor size ≥7 mm based on preoperative US. CLND need be considered in cN0 patients who are men, aged <50 years or tumor size ≥7 mm based on preoperative US.

## Background

Thyroid cancer is the most frequent endocrine malignancy, accounting for approximately 1% of all malignant tumors in the United States [1] and 5.90% (with the incidence of 21.76/100,000) in one district of Wenzhou (a coastal city in Southeast China, with a referral population of 7,558,000) [2]. Papillary thyroid carcinoma (PTC) represents the most common type, with incidence increasing each year, especially in papillary thyroid microcarcinoma (PTMC) which is a type of PTC with a maximum diameter of 1.0 cm or less [3]. Unfortunately PTMC treatment is still controversial. While some doctors believe that the observation is appropriate, most advocate surgical resection [4,5]. In general, TT or therapeutic CLND should be performed when bilateral lobe involvement or CLNM is preoperatively detected. However, unilateral lobectomy may be applicable for patients who have only found PTMC confined to the unilateral lobe based on preoperative detection [6-8]. It had been reported that the rate of PTMC bilateral involvement was about 10–30% [9-11] and the rate of CLNM could reach up to 61% [12]. Whether TT or CLND should be offered to patients who could not detect bilateral lobe involvement or CLNM preoperatively is still controversial. So, how to predict PTMC with bilateral involvement or CLNM is crucial for determining initial surgical resection.

In this study, we retrospectively examined the incidence and related risk factors of PTMC with bilateral involvement and CLNM, attempting to reveal the independent predictors and hope to identify a subset of PTMC patients who may benefit from TT or CLND in initial surgery.

## Methods

This study was approved by the institutional review board of the First Affiliated Hospital of Wenzhou Medical College. A total of 211 cases of PTMC patients were initially treated in our oncology department between 2010 and 2011. All patients had no history of neck irradiation and underwent preoperative US and US-guided fine needle aspiration biology (FNAB) whose diagnosis was PTC. TT was routinely operated and ultimately diagnosed as PTMC. Cases with accidental discovery of PTC and patients who underwent completion of total thyroidectomy were not found in our series. All patients accepted CLND routinely; apart from 89 patients whose preoperative detection of suspicious neck lymph nodes, 122 cases with cN0 who underwent prophylactic CLND were included in the study of CLNM.

The following variables were used to analyze risk factors of PTMC bilateral involvement and CLNM: gender, age at diagnosis, final pathological primary tumor size and mulifocality, extrathyroidal extension (ETE), with Hashimoto thyroiditis (HT), primary tumor size and mulifocality based on preoperative US. Final pathological PTMC bilateral involvement and CLNM were examined as binary variables. All pathological findings were confirmed by two experienced endocrine pathologists and PTMC diagnosis was defined as PTC whose maximum size is ≤1 cm according to World Health Organization standards. Bilateral PTMC was diagnosed according to PTMC in both sides of the thyroid gland which differed from unilateral PTMC with foci in only one side lobe or isthmus. Mulifocality was defined as more than one tumor lesion in the primary tumor ipsilateral lobe. Tumor size in multifocal PTMC patients was measured according to maximum diameter of the primary tumor. Primary tumor size based on preoperative US was measured by the standard of change in echo edge. Preoperative mulifocality was defined as more than one suspicious lesion in one side lobe according to US findings. Suspicious lesions based on US expressed as irregular shape, ill-defined margin, echogenicity, micro-calcification, or vascularity.

The results are expressed as the mean ± SD. To compare the differences, two-tailed student *t* test was used in measurement data and chi-square test or Fisher exact test was used in enumeration data, while Logistic regression multivariate analysis was used for further study. The application of two-tailed test *P* value <0.05 was considered statistically significant. Statistical analysis was performed using SPSS 18.0.

## Results

A total of 211 patients consisted of 179 women and 32 men with a median age of 49 ± 11 years (range, 23–79 years). The median size of the primary thyroid cancer was 5.8 ± 2.7 mm (1–10 mm). Mulifocality was found in 25 patients (11.8%) and HT was found in 59 patients. ETE was found in 10 patients (4.7%), in which seven cases were classified as T3 staging and three cases were classified as T4 staging (Table 1). Of the 211 patients with PTMC, 54 (25.6%) had bilateral disease, and 157 (74.4%) were unilateral PTMCs. Univariate analysis of potential clinicopathologic factors associated with bilateral PTMC is shown in Table 1. Contralateral PTMC was significantly more frequent in patients with final pathological multifocality (*P* <0.001) and primary tumor size ≥5 mm or ≥7 mm (*P* = 0.015, 0.018, respectively). There were no significant differences between the presence of contralateral carcinoma and age, gender, ETE, T stage and with HT. We also found the preoperative multifocality (*P* <0.001) and tumor size (≥5 mm or ≥7 mm, *P* = 0.013, 0.020, respectively) which prompted by US were associated with bilateral PTMC.

**Table 1:** Clinicopathological characteristics and univariate analysis of 211 patients

**Characteristics**	**Total (*n*** **= 211)**	**Bilateral PTMC ** **(*n*** **= 54)**	**Unilateral PTMC ** **(*n*** **= 157)**	***P* value**
Gender(M/F)	32/179	8/46	24/133	0.934
*Age ± SD (years)*	49 ± 11	49 ± 10	48 ± 11	0.679^a^
≥45/<45	143/68	38/16	105/52	0.636
≥50/<50	97/114	23/31	74/83	0.564
*Primary tumor size ± SD (mm)*	5.8 ± 2.7	6.8 ± 2.6	5.5 ± 2.7	0.001^a,b^
≥5/<5	144/67	44/10	100/57	0.015 ^b^
≥7/<7	81/130	28/26	53/104	0.018 ^b^
Multifocality (%)	25 (11.8)	21 (38.9)	4 (2.6)	<0.001^b^
ETE (%)	10 (4.7)	5 (9.3)	5 (3.2)	0.128^c^
T stage (T1/2:T3/4)	201/10	49/5	152/5	0.128^c^
With HT (%)	59 (28.0)	20 (37.0)	39 (24.8)	0.085
*Preoperative tumor size (mm)*	6.1 ± 2.9	7.1 ± 2.7	5.8 ± 2.8	0.005^a,b^
≥5/<5	152/59	46/8	106/51	0.013 ^b^
≥7/<7	85/126	29/25	56/101	0.020 ^b^
Preoperative multifocality (%)	19 (8.6)	16 (29.6)	3 (1.9)	<0.001^b,c^

^a^Calculated using two-tailed student *t* test.

^b^Statistically significant (P <0.05).

^c^Calculated using Fisher exact test, others calculated using chi-square test.

ETE, extrathyroidal extension; HT,Hashimoto thyroiditis; PTMC, papillary thyroid microcarcinoma.

We conducted multivariate logistic regression analysis of final pathological factors and preoperative factors separately. The results showed that tumor size ≥5 mm as the size threshold was not an independent factor, whose results of the analysis did not show. However, final pathological multifocality (*P* <0.001, OR = 29.657) and primary tumor size ≥7 mm (*P* = 0.005, OR = 2.869) still showed a significant correlation with bilateral PTMC. The same results were found in preoperative multifocality (*P* < 0.001) and primary tumor size ≥7 mm (*P* = 0.014, OR = 2.398) which prompted by US. Table 2 shows the results of logistic regression analysis.

**Table 2:** Logistic regression analysis of the significant risk factors in association with bilateral PTMC

**Factors**	**β**	**S.E.**	***P* value**	**OR value**	**95% CI**
*Final pathological factors*					
Tumor size (≥7 mm/<7 mm)	1.054	0.378	0.005^a^	2.869	1.366–6.023
Multifocality	3.390	0.601	<0.001^a^	29.657	9.140–96.234
Constant	−2.028	0.286			
*Preoperative factors*					
Preoperative tumor size(≥ 7 mm/<7 mm)	0.875	0.357	0.014^a^	2.398	1.192–4.824
Preoperative multifocality	3.174	0.668	<0.001^a^	23.900	6.456–88.475
Constant	−1.806	0.263			

^a^Statistically significant (*P* <0.05).

Of 122 cN0 patients who underwent CLND, 60 cases (49.2%) had CLNM. Univariate analysis of relevant clinicopathologic factors in association with CLNM is expressed in Table 3. Apart from ETE, T stage and with HT, others as men (*P* = 0.015), aged < 50 years (*P* = 0.011), final pathological tumor size ≥ 7 mm (*P* = 0.011), multifocality (*P* = 0.020), and bilateral PTMC (*P* = 0.020) were significantly related to CLNM. Similarly, preoperative tumor size ≥ 7 mm and multifocality which was promoted by US were also significant differences between the central lymph node positive and negative groups (*P* = 0.001 and 0.027, respectively).

**Table 3:** Clinicopathological characteristics and univariate analysis of 122 cases of PTMC patients who underwent prophylactic CLND

**Characteristics**	**Total (*n*** **= 122)**	**CLNM + (*n*** **= 60)**	**CLNM - (*n*** **= 62)**	***P* Value**
Gender (M/F)	60/62	44/16	16/46	0.015^a^
*Age ± SD (years)*	48 ± 11	46 ± 10	50 ± 11	0.044^a,b^
≥45/<45	83/39	37/23	46/16	0.138
≥50/<50	57/65	21/39	36/26	0.011^a^
*Tumor size ± SD (mm)*	6.5 ± 2.6	7.2 ± 2.3	5.8 ± 2.7	0.002^a,b^
≥5/<5	95/27	51/9	44/18	0.062
≥7/<7	59/63	36/24	23/39	0.011^a^
Multifocality (%)	19 (15.6)	14 (23.3)	5 (8.1)	0.020^a^
ETE (%)	7 (5.7)	5 (8.3)	2 (3.2)	0.269^c^
T stage (T1/2:T3/4)	115/7	55/5	60/2	0.269^c^
With HT (%)	43 (35.2)	19 (31.7)	24 (38.7)	0.416
Bilateral PTMC (%)	60 (50.8)	26 (61.9)	34 (42.5)	0.042^a^
*Preoperative tumor size (mm)*	6.8 ± 2.8	7.5 ± 2.6	6.1 ± 2.9	0.008^a^
≥5/<5	99/23	52/8	47/15	0.125
≥7/<7	61/61	37/23	24/38	0.001^a^
Preoperative multifocality (%)	16(13.1)	12(20.0)	4(6.6)	0.027^a^

^a^ Statistically significant (*P* < 0.05).

^b^Calculated using two-tailed student *t* test.

^c^Calculated using Fisher exact test, others calculated using chi-square test.

CLNM, central lymph node metastasis; ETE, extrathyroidal extension; HT, Hashimoto thyroiditis; PTMC, papillary thyroid microcarcinoma.

However, further multivariate analysis showed that bilateral PTMC did not enter the final logistic regression equation (*P* = 0.367) and multifocality was also a confounding factor (*P* = 0.138). Interestingly, being male (*P* = 0.008, OR = 4.848), aged <50 years (*P* = 0.004, OR = 3.442), and the final pathological tumor size ≥ 7 mm (*P* = 0.011, OR = 2.908) were independently associated with CLNM. At the same time, logistic regression analysis of preoperative factors also reached a similar result: males (*P* = 0.008, OR = 4.910), aged <50 years (*P* = 0.005, OR = 3.311) and the preoperative tumor size ≥ 7 mm (*P* = 0.009, OR = 2.964) based on US were independent predictors of CLNM. Table 4 shows results of logistic regression analysis using the significant risk factors in association with CLNM.

**Table 4:** Logistic regression analysis of the significant risk factors in association with CLNM

**Factors**	**β**	**S.E.**	***P* value**	**OR value**	**95% CI**
*Final pathological factors*					
Gender (M/F)	1.578	0.592	0.008 ^a^	4.848	1.519–15.466
Age (<50y/≥50y)	1.236	0.428	0.004^a^	3.442	1.488–7.963
Tumor size (≥7 mm/<7 mm)	1.067	0.419	0.011^a^	2.908	1.278–6.614
Multifocality	0.991	0.667	0.138	2.693	0.728–9.956
Bilateral PTMC	0.436	0.484	0.367	1.547	0.600–3.993
Constant	−1.787	0.448			
*Preoperative factors*					
Gender (M/F)	1.591	0.596	0.008^a^	4.910	1.526–15.796
Age (<50y/≥50y)	1.199	0.424	0.005^a^	3.311	1.443–7.634
Preoperative tumor size (≥ 7 mm/<7 mm)	1.086	0.418	0.009^a^	2.964	1.305–6.729
Preoperative multifocality	0.806	0.706	0.253	2.240	0.562–8.931
Bilateral PTMC	0.584	0.466	0.210	1.793	0.719–4.469
Constant	−0.600	0.371			

^a^Statistically significant (*P* < 0.05).

## Discussion

PTMC will likely draw continued attention given its increasing incidence in recent years. Although TT and CLND currently constitute the common initial surgical management of patients with larger PTC, the best surgical extent for PTMC is still a controversial topic [13]. When surgery is performed for PTMC, it is generally accepted that TT or CLND should be performed for preoperatively detected bilateral PTMC or CLNM. However, the optimal extent of surgical resection in cases with preoperatively undetected bilateral PTMC or CLNM remains a topic of debate. We retrospectively reviewed clinicopathological factors of PTMC patients and found that primary tumor ≥ 7 mm and multifocality were independent predictors of bilateral PTMC, and being male, aged <50 years, and primary tumor ≥ 7 mm could be used to predict CLNM. Further, multifocality and primary tumor ≥ 7 mm could be prompted by preoperative US to identify PTMC patients who need TT or CLNM.

Surgeons who advocated TT believed that thyroid cancer often presented in both sides of thyroid lobes. Then, how many cases had bilateral involvement in thyroid cancer patients? Studies had reported that the rate of bilateral PTC ranged from13% to 56% that could be found in completion thyroidectomy and TT [14-17] and incidence of bilateral PTMC was approximately 10–30% in PTMC patients [9-11,17,18]. In our study, 25.6% of PTMC patients who underwent TT had contralateral PTMC. This rate was consistent with the previous reports [9-11,17,18]. In our opinion, a 25.6% ratio for a contralateral carcinoma does not justify routine TT for all PTMC patients. Multifocality had been prompted as a predictor of contralateral thyroid cancer in a number of studies [14,15,19,20]. Our study confirmed the predictive value of multifocality and found that 84% of multifocal PTMC patients had contralateral lobe foci, which may be due to that bilateral PTMC often arise from a single clone and intrathyroidal metastasis [21]. Primary tumor size in the previous study was considered to be nothing to do with the contralateral PTC [15,20,22-24]. However, our study found that bilateral PTMC may be more vulnerable to have a large primary tumor (≥ 5 mm/≥ 7 mm), and primary tumor ≥ 7 mm was an independent predictor of contralateral lobe PTMC. A reason inconsistent results may be attributed to our research was a retrospective study and the object was PTMC patents who underwent TT, compared to the previous prospective study [22] and patients with clinical unilateral PTMC [20]. Moreover, the number of PTMC cases in our study increased compared to the study of Pitt *et al*. [15] and we firstly used a tumor size >7 mm as the size threshold in study of bilateral PTMC. In addition, previous studies had reported that bilateral PTC was inter-related with advanced T stage and high incidence of ETE [16,23]. However, we did not obtain the same results in PTMC cases agreed with previous studies of bilateral PTMC [20,22]. The reasons may be attributed to that PTMC was found early and micro-foci, which may not yet demonstrate full biological behavior. Others such as gender, age, and with HT were not found significant difference as other papers reported [20,22,24].

Therapeutic CLND in PTMC patients is always necessary, whereas prophylactic CLND remains a subject of considerable debate. The 2009 guidelines of the American Thyroid Association (ATA) recommend prophylactic CLND in patients with cN0 PTCs, particularly in those with advanced T3 and T4 primary tumors, but do not recommend elective central neck dissection in patients with small, non-invasive cN0 PTCs [25]. A high incidence of CLNM in PTMC patients has been reported (up to 61%) [12]. Our study found the CLNM rate was 49.2% in PTMC patients with preoperatively undetected CLNM. Although US has been regarded as a sensitive imaging modality for thyroid screening and diagnosis, US has a low sensitivity in evaluation of metastasis in CLNM [26]. Similarly, in our study, imaging studies were not useful in the detection of CLNM. Age is known to be an important prognostic factor for patients with PTC greater than 1 cm; however, its prognostic value in PTMC was uncertain [12,27-29]. Most studies used an age <45 years as the age threshold [30,31]. In our study, age <50 years was found to be independently associated with the greatest risk of CLNM, but an age <45 years as the age threshold may be too small to assess the CLNM. In addition, consistent with previous reports, we also found that being male was an independent risk factor of CLNM. Previous studies have suggested that CLNM was associated with tumor size [12,32,33]. Most studies used a tumor size >5 mm as the size threshold. Lee *et al*. thought that a cutoff value of 7 mm may be considered the threshold of aggressiveness of PTMCs [34]. Our analysis found that tumor size ≥ 7 mm was independently associated with CLNM, but a threshold >5 mm may be too small to assess the CLNM. We also revealed preoperative tumor size ≥ 7 mm based on US could be used to predict CLNM. Thus, preoperative tumor size ≥ 7 mm based on US, combined with being male and aged < 50 years can be used to predict the CLNM, and guide CLND. In our study, we found that cases who had bilateral PTMC and multifocality may be prone to CLNM (*P* = 0.042 and 0.020, respectively), but they were not independent risk factors. In addition, disagreeing with a previous report [28], our study did not prompt that CLNM was associated with ETE and T stage. The reason may be due to our low rate of ETE which was not included microscopic extension.

Although our analysis is limited because of the lack of both long-term follow-up results and prognostic implication of bilateral PTMC and CLNM, we did highlight the predictive value of preoperative primary tumors ≥ 7 mm and multifocality both prompted by US, which can be contributed to compensate for the lack of preoperative sensitivity to reveal undetected bilateral PTMC and CLNM.

## Conclusions

Of all PTMC patients who underwent TT, 25.6% had bilateral PTMC. Tumor size ≥ 7 mm and at least one multifocal lobe were independent risk factors for bilateral PTMC and both can be prompted by US to guide TT. The rate of CLNM was 49.1% in cN0 patients. Factors such as tumor foci ≥7 mm based on final pathology or US, being male, and a younger age (<50 years) can be used to predict CLN metastasis. Surgeons are justified to offer TT to PTMC patients with multifocality and foci ≥7 mm based on preoperative US. CLND needs to be considered in PTMC patients who are men, aged < 50 years, or foci ≥7 mm based on preoperative US.

## Competing interests

The author(s) declare that they have no competing interest.

## Authors’ contributions

YLZ substantially contributed to the conception and design, acquisition of data, drafting and revision of the article. ELG and WZ participated in the data collection and revisions; HY and GLG substantially contributed to analysis and interpretation of data, revision of the article. XHZ and OCW acted as corresponding authors and did the conception and revisions. All authors read and approved the final manuscript.
